# Supplementary material for: Aurora A kinase activation contributes to the fibrotic phenotype in systemic sclerosis through primary cilia shortening
Source: Arthritis Res Ther. 2026 May 7;28:134. doi: 10.1186/s13075-026-03826-6 (PMC13317384; doi:10.1186/s13075-026-03826-6)
Supplement: Supplementary file 1 — Supplementary Material 1. [file 13075_2026_3826_MOESM1_ESM.pptx]

## Slide 1
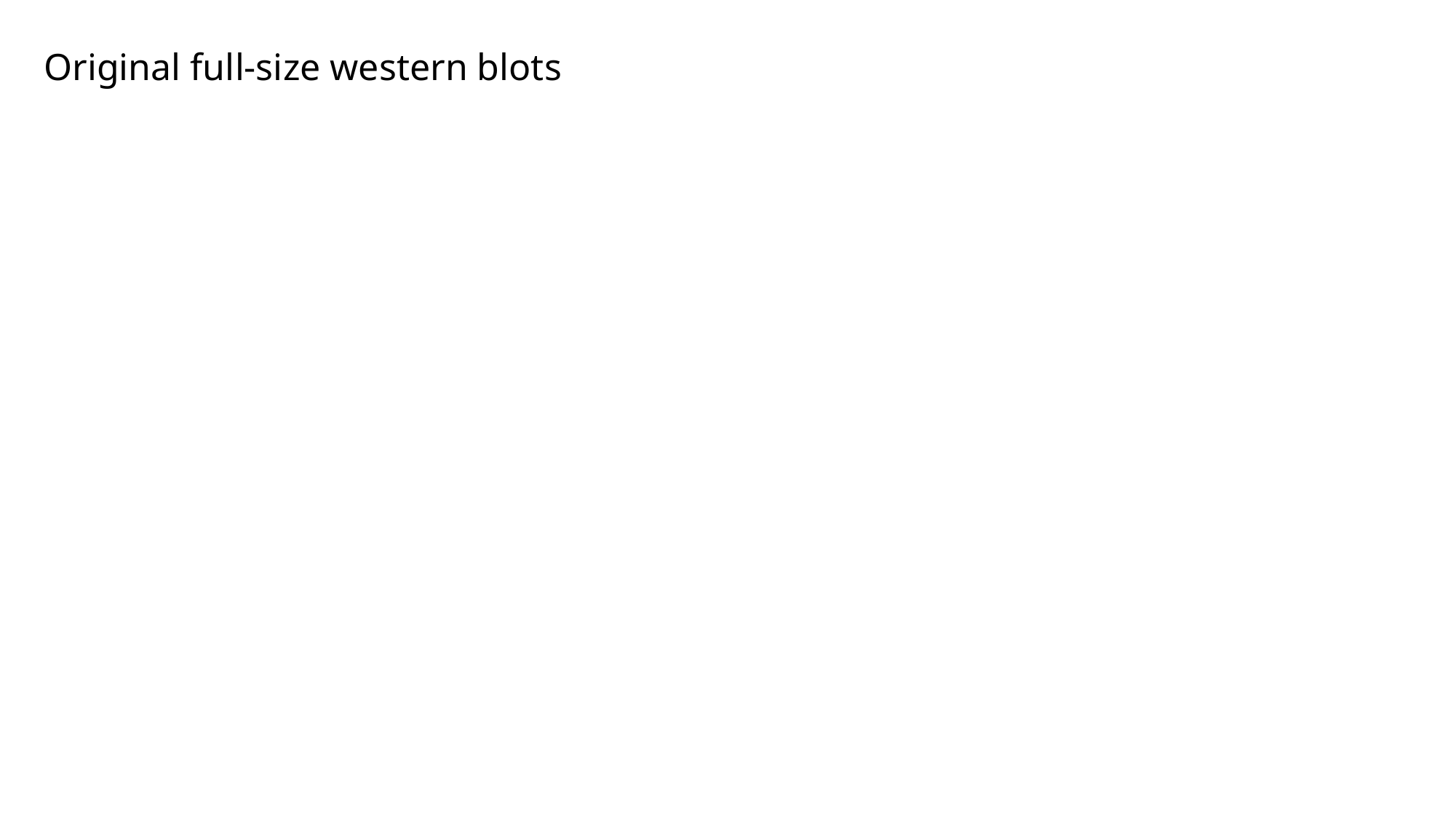

# Original full-size western blots

## Slide 2
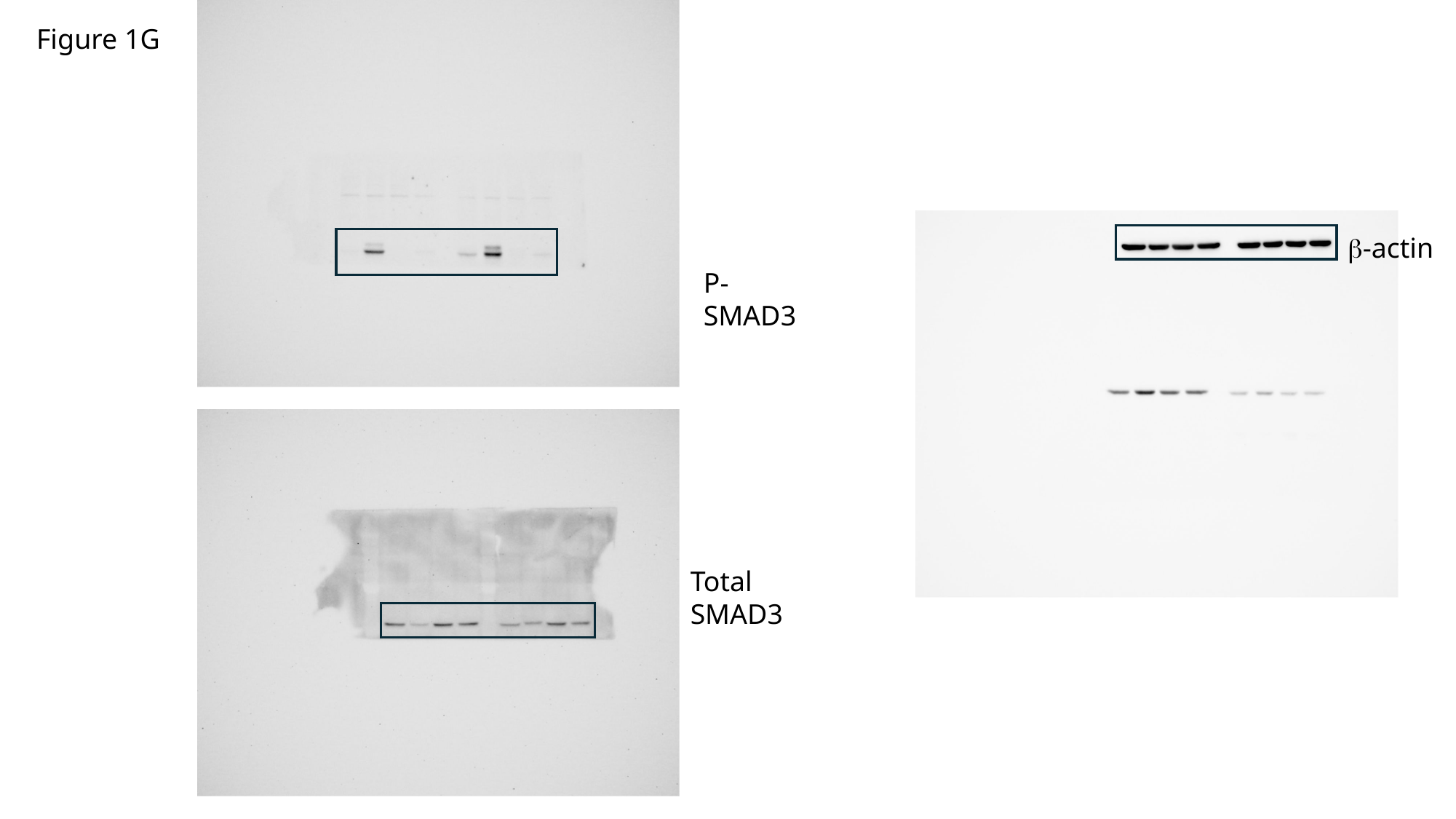

Figure 1G
b-actin
P-SMAD3
Total SMAD3

## Slide 3
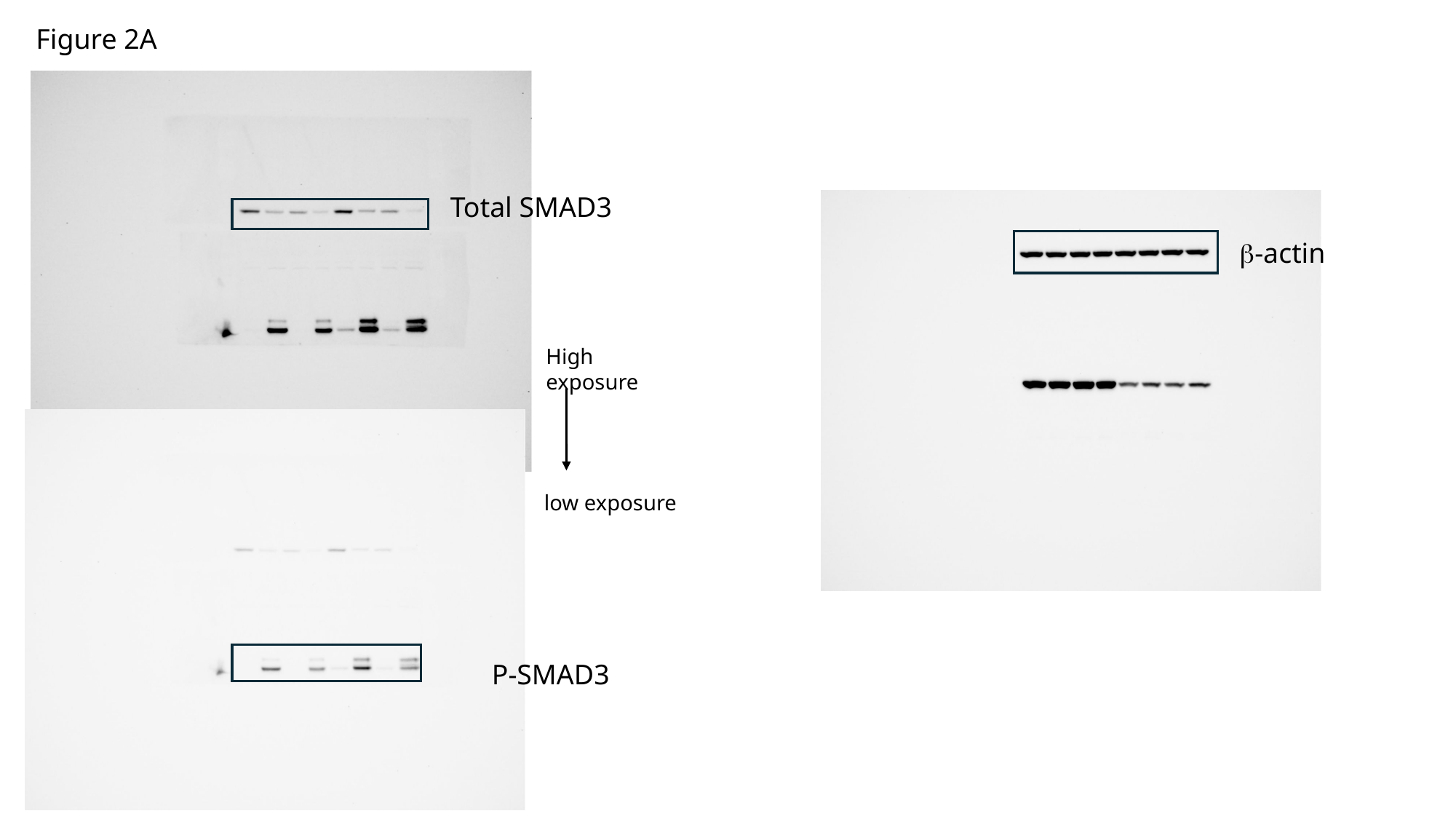

Figure 2A
Total SMAD3
b-actin
High exposure
low exposure
P-SMAD3

## Slide 4
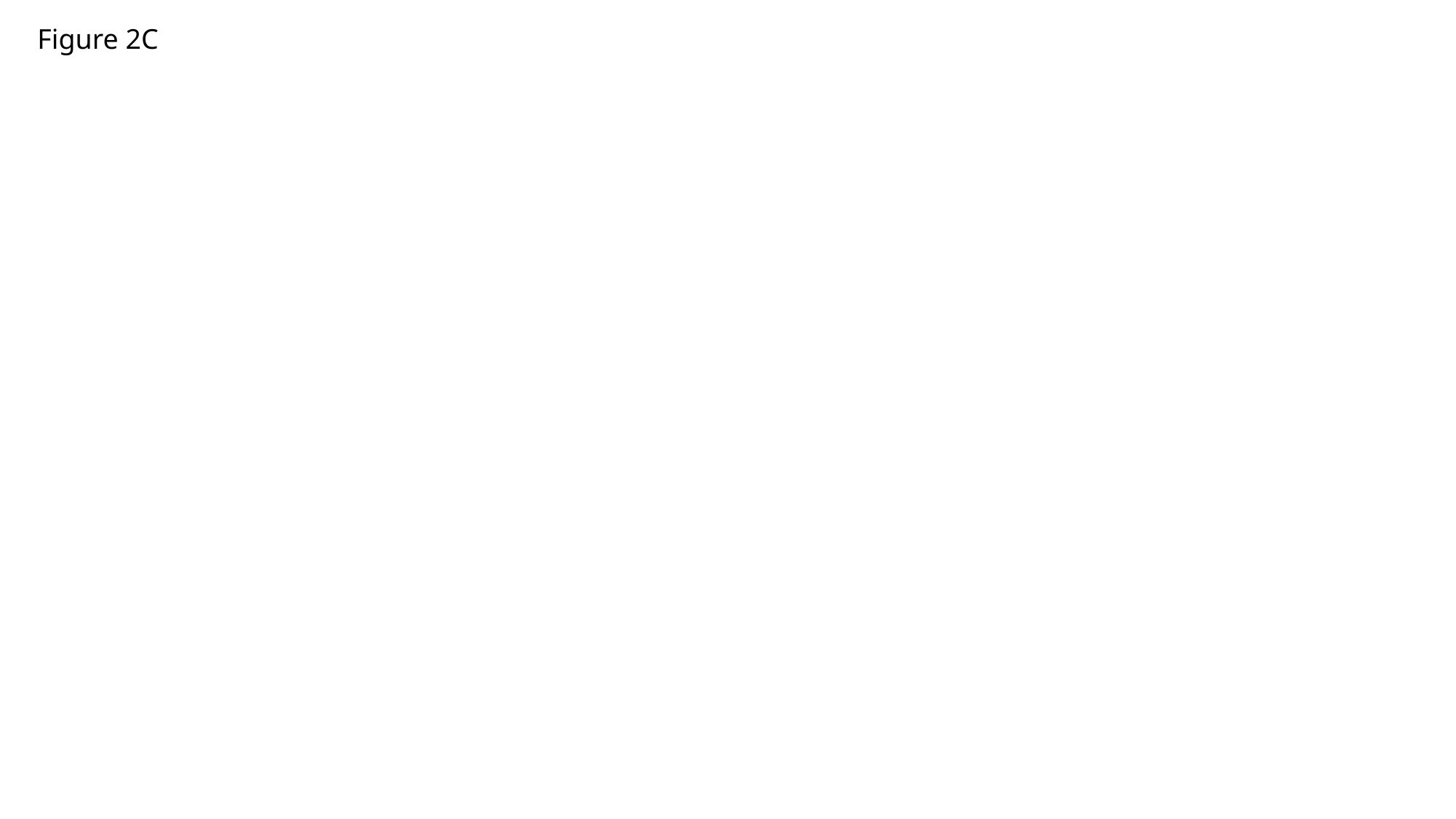

Figure 2C

## Slide 5
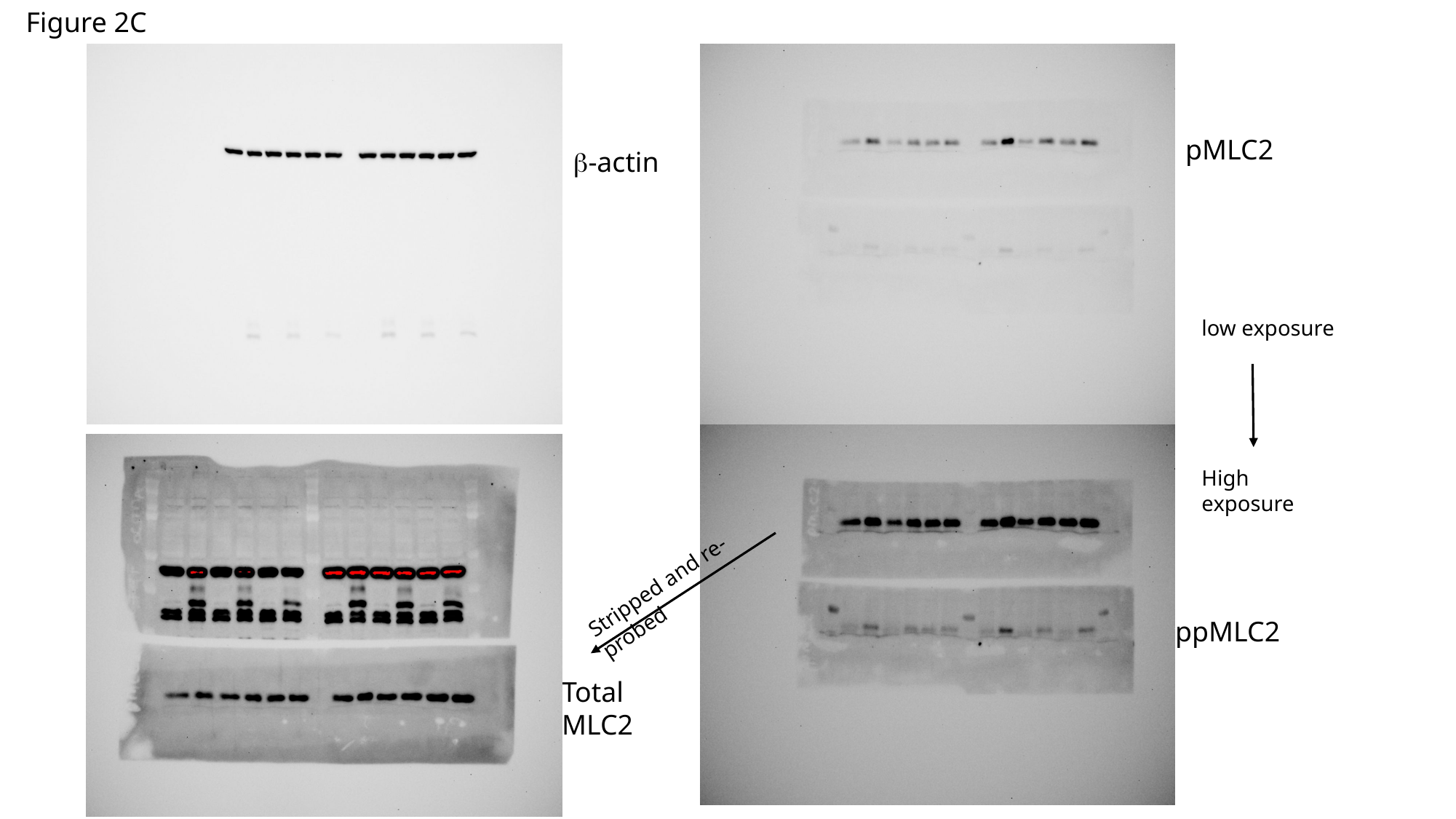

Figure 2C
pMLC2
b-actin
low exposure
High exposure
Stripped and re-probed
ppMLC2
Total MLC2

## Slide 6
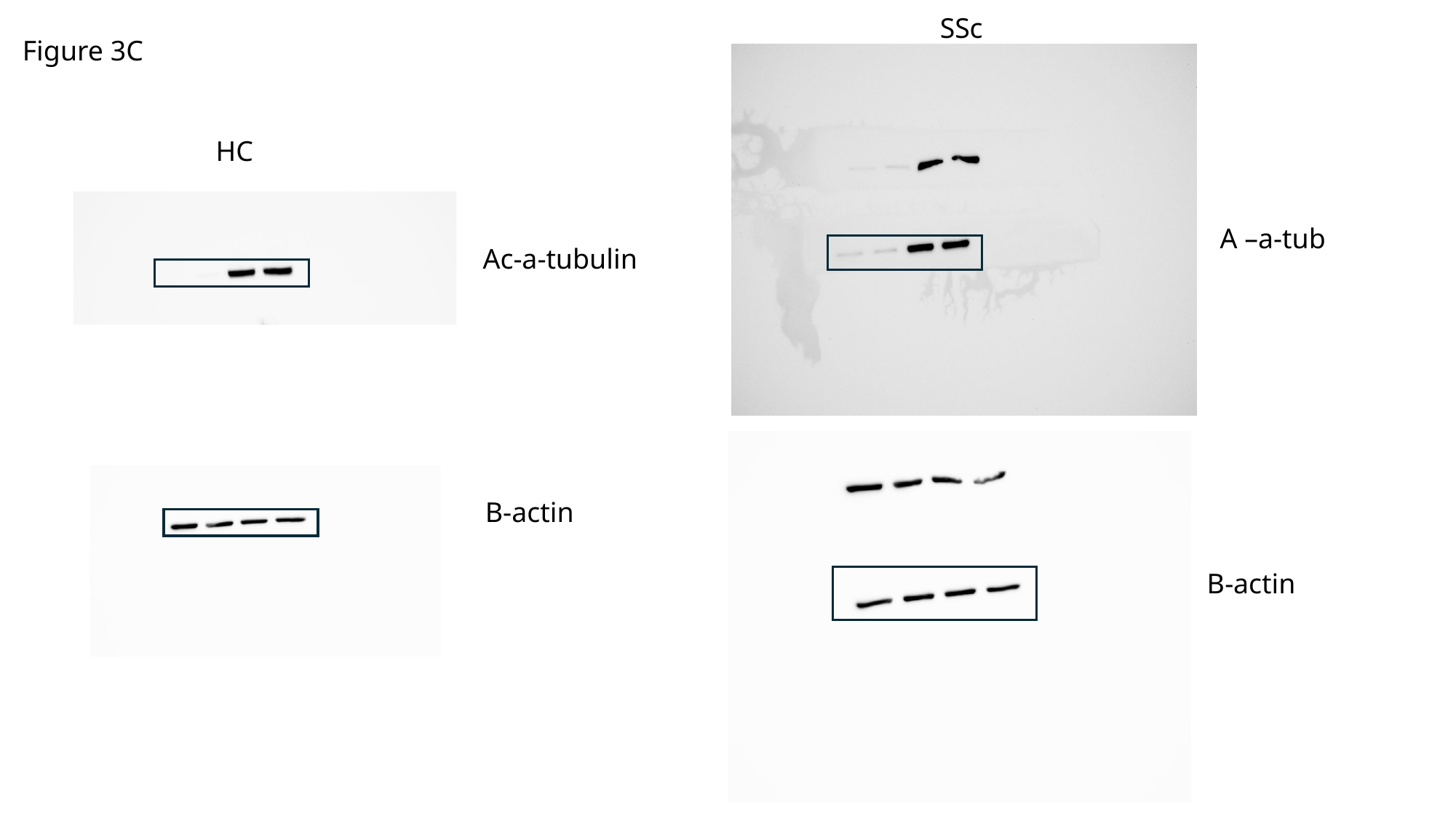

SSc
Figure 3C
HC
A –a-tub
Ac-a-tubulin
B-actin
B-actin

## Slide 7
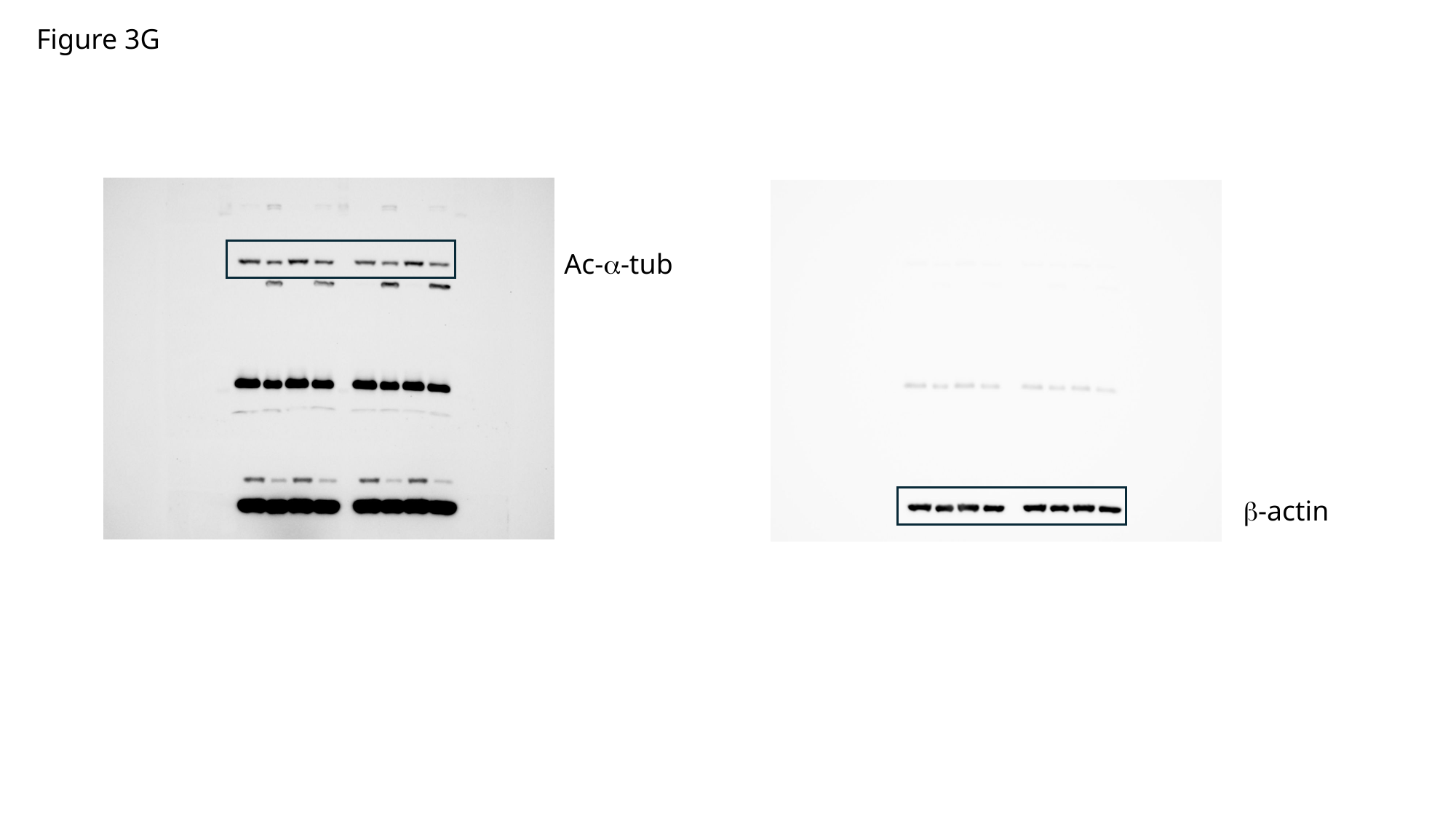

Figure 3G
Ac-a-tub
b-actin

## Slide 8
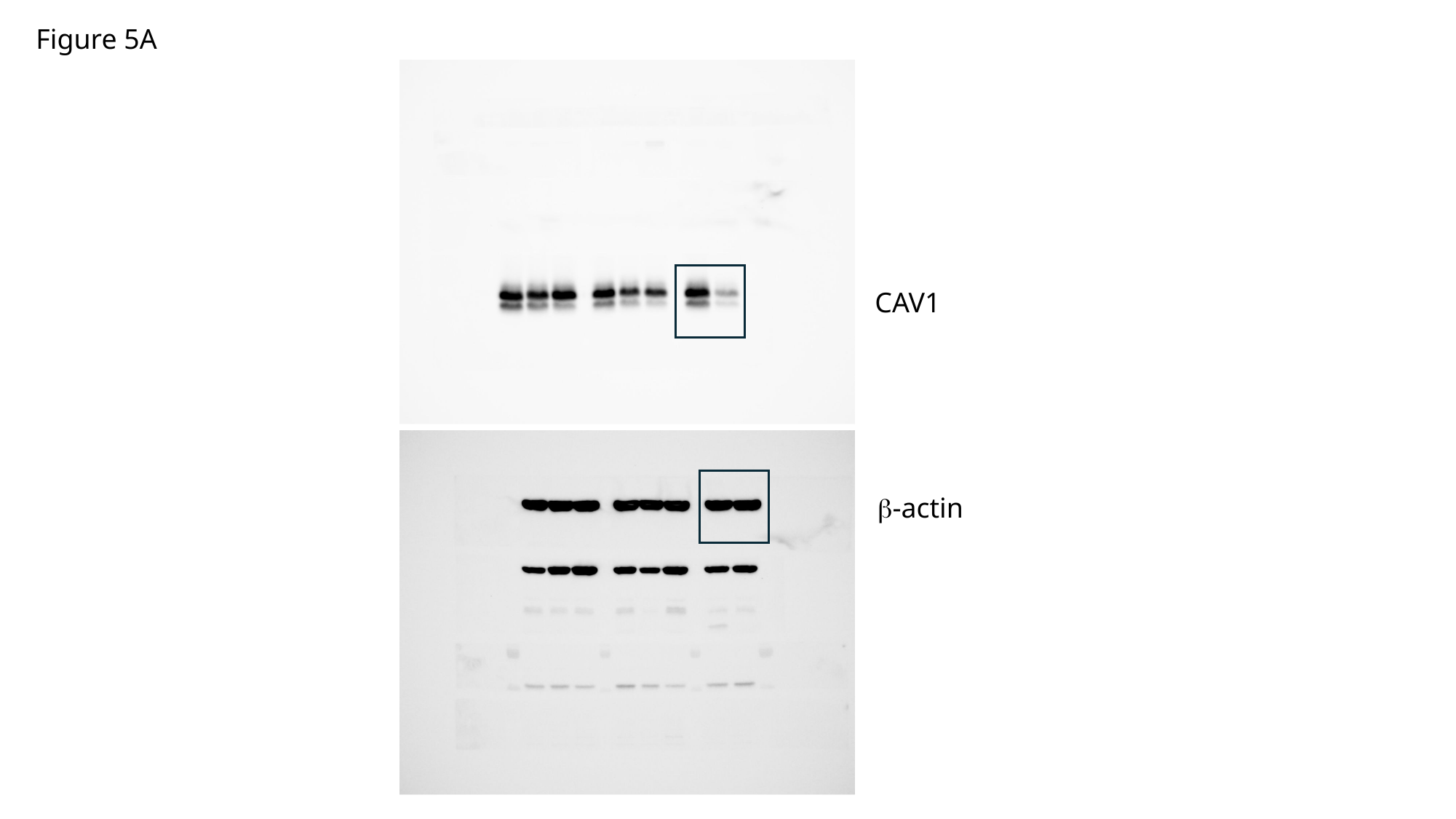

Figure 5A
CAV1
b-actin

## Slide 9
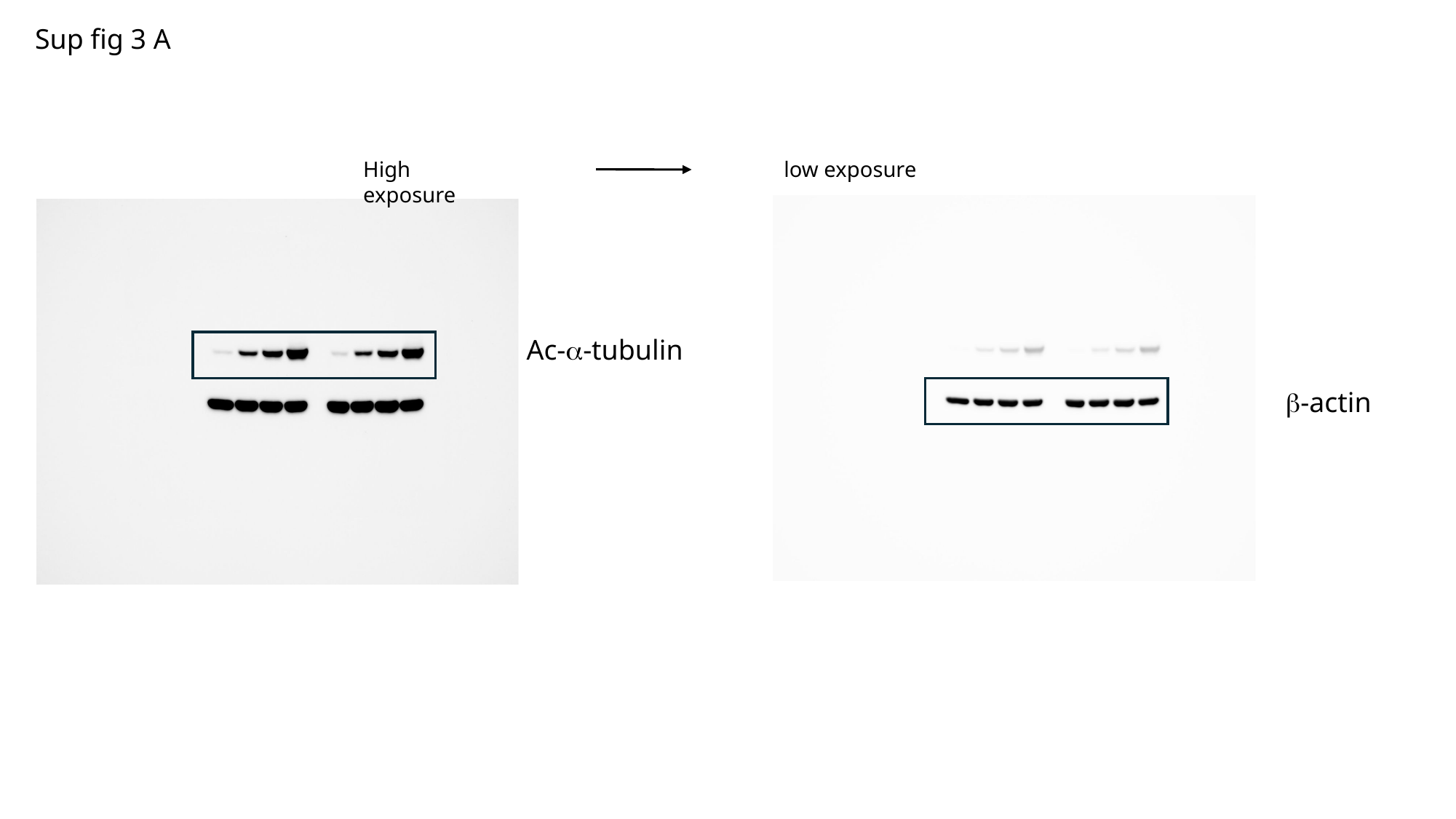

Sup fig 3 A
High exposure
low exposure
Ac-a-tubulin
b-actin
